# Metabolic Response in Rats following Electroacupuncture or Moxibustion Stimulation

**DOI:** 10.1155/2019/6947471

**Published:** 2019-05-02

**Authors:** Jingjing Xu, Xianwei Lin, Kian-Kai Cheng, Huan Zhong, Mi Liu, Guoshan Zhang, Guiping Shen, Jiyang Dong

**Affiliations:** ^1^Department of Electronic Science, Fujian Provincial Key Laboratory for Plasma and Magnetic Resonance, Xiamen University, Xiamen 361005, China; ^2^Innovation Centre in Agritechnology, Universiti Teknologi Malaysia, 84600 Muar, Johor, Malaysia; ^3^College of Acupuncture and Moxibustion, Hunan University of Chinese Medicine, Changsha 410007, China

## Abstract

Electroacupuncture and moxibustion are traditional Chinese medicine practices that exert therapeutic effects through stimulation of specific meridian acupoints. However, the biological basis of the therapies has been difficult to establish; thus the current practices still rely on ancient TCM references. Here, we used a rat model to study perturbations in cortex, liver, and stomach metabolome and plasma hormones following electroacupuncture or moxibustion treatment on either stomach meridian or gallbladder meridian acupoints. All treatment groups, regardless of meridian and mode of treatment, showed perturbation in cortex metabolome and increased phenylalanine, tyrosine, and branched-chain amino acids in liver. In addition, electroacupuncture was found to increase ATP in cortex, creatine, and dimethylglycine in stomach and GABA in liver. On the other hand, moxibustion increased plasma enkephalin concentration, as well as betaine and fumarate concentrations in stomach. Furthermore, we had observed meridian-specific changes including increased N-acetyl-aspartate in liver and 3-hydroxybutyrate in stomach for gallbladder meridian stimulation and increased noradrenaline concentration in blood plasma following stimulation on stomach meridian. In summary, the current findings may provide insight into the metabolic basis of electroacupuncture and moxibustion, which may contribute towards new application of acupoint stimulation.

## 1. Introduction 

In traditional Chinese medicine (TCM), acupuncture and moxibustion play vital roles in treatment of diseases and promotion of health [1–3]. Their therapeutic effects are exerted through stimulation of specific meridian acupoints, either by using fine needles (acupuncture) or by heat from burning moxa wool (moxibustion).

Compared with conventional acupuncture practice, electroacupuncture involves insertion of fine needles together with an electrical stimulation. Multiple studies have shown that electroacupuncture produces more consistent and reproducible results in both clinical and research studies and is found effective for pain control [4], functional tissue recovery [5], and immunomodulation [6]. On the other hand, moxibustion can be conducted in two different ways (i.e., direct and indirect methods), depending on whether the moxa wool has direct or indirect contact with skin. The indirect moxibustion method (as was used in the present study) is known to exert biophylactic effects such as anti-inflammatory activity [7], downregulation of heat shock protein expression [8], and reduction in lipid peroxide level [9].

Despite an increased interest in TCM interventions, the mechanisms of action of electroacupuncture and moxibustion are not fully understood [10, 11]. Most of the electroacupuncture and moxibustion studies involved patients and animal disease models, whereas the metabolic responses of healthy individuals following the interventions are not established. The study on healthy individuals may be crucial in order to understand the effects of electroacupuncture and moxibustion in different pathological states.

Previously, Quan and colleagues mapped the molecular underpinning of TCM by using ‘omics' technology and network representations [12]. Among the ‘omics' platforms, metabolomics is a high-throughput profiling technique that can monitor metabolic changes caused by interventions [13, 14], which may serve as an excellent platform to investigate metabolic mechanism of electroacupuncture and moxibustion [15–17].

In previous reports, electroacupuncture treatment on acupoints along the stomach meridian (SM) has been found effective in enhancing gastrointestinal motility, improving gastric mucosal blood flow, and protecting gastric mucosa from injury [18, 19]. In addition, acupuncture treatment on acupoints along the gallbladder meridian (GM) was found beneficial in promoting bile production and secretion, improving insomnia, and alleviating migraines [20, 21]. In the current study, these two specific meridians were selected as two studying cases to investigate the metabolic changes perturbed by electroacupuncture or moxibustion intervention. Briefly, tissue extracts (i.e., cortex, stomach, and liver) from healthy rats receiving either electroacupuncture or moxibustion stimulation were examined by a combination of nuclear magnetic resonance (NMR) spectroscopy and multivariate statistical analysis. The scope of this work was to probe into metabolic basis of these two traditional interventions using NMR-based metabolomics and contribute towards a better understanding of theoretical foundation for the therapies.

## 2. Methods and Materials

### 2.1. Ethical Statement

Animal care and experimental procedures used in the current study were approved by the Institutional Animal Care and Use Committee of Hunan University of Chinese Medicine (Ethics No. SCXK 2011-0003). This study was carried out adhering to guidelines provided by National Institutes of Health for the Care and Use of Laboratory Animals and all efforts were made to minimize suffering of animals.

### 2.2. Animal Handling

In this study, sixty healthy male SD rats (150±20 g weight, eight weeks old) were housed individually in metabolism cages, and the animals were provided with food and water* ad libitum*. The animal room was under controlled condition (temperature, humidity, and a 12-hour light-dark cycle). After acclimatization for one week, all rats were randomly divided into five groups (*n* = 12): (1) NC group: normal control rats without treatment, (2) EASM group: rats with electroacupuncture stimulation on SM acupoints, (3) MBSM group: rats with moxibustion stimulation on SM acupoints, (4) EAGM group: rats with electroacupuncture stimulation on GM acupoints, and (5) MBGM group: rats with moxibustion stimulation on GM acupoints.

### 2.3. Electroacupuncture and Moxibustion Treatment

The four treatment groups were subjected to either electroacupuncture or moxibustion treatment (a 20-minute session per day) for two weeks. In order to minimize variability due to handling, only one acupuncturist with more than 10 years of acupuncture and moxibustion experience conducted the treatment procedure throughout the study. The acupuncturist is a member of the College of Acupuncture and Moxibustion, Hunan University of Chinese Medicine, and is qualified as a medical practitioner acupuncturist by the National Health and Family Planning Commission of China.

**Figure 1:**
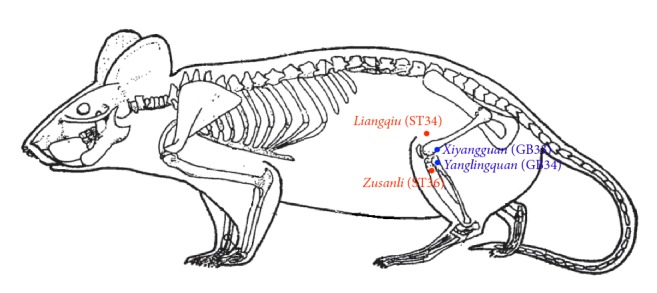
Points selected for electroacupuncture and moxibustion treatments.

For treatment on the stomach meridian, the* Zusanli* (ST36) and* Liangqiu* (ST34) (Figure 1) acupoints along the meridian were selected. Previously, stimulation on these acupoints was found to ameliorate gastrointestinal disorders [22]. On the other hand, the* Yanglingquan* (GB34) and* Xiyangguan* (GB33) acupoints were selected for treatment on the gallbladder meridian. The locations of acupoints were determined according to the Government Channel and Points Standard GB12346-90 of China and “The Veterinary Acupuncture of China.”

*Electroacupuncture intervention*: two-channel electrical stimulations were performed with a pulse generator (Model G6805-2; Qingdao Xinsheng Medical Instrument Factory, Shandong, China) via stainless-steel acupuncture needles of 0.25 mm in diameter. The electrical stimuli consisted of intermittent and irregular waves (intermittent wave: 4 Hz; irregular wave: 50 Hz) with voltage ranging from 2 to 4 V. The electrical intensity was just strong enough to induce slight twitches in the hind limbs of the animals.

*Moxibustion intervention*: rats from the MBSM and MBGM groups were subjected to indirect moxibustion using a commercially available moxibustion box. The box had a cylindrical opening to hold a pillar of moxa stick and an elastic cord to affix it to acupoints. The moxa stick (Φ18 × 200 mm) was wrapped with moxa floss, which is white, soft, cotton-like fiber prepared from moxa leaves. All rats received the same fixation and hair located around the acupoints was cut off to expose the local skin before treatment. The devices were adjusted until rats were comfortable and stopped struggling. Burning injury was carefully avoided in the process by the acupuncturist by whisking away the burning ash in a timely manner.

### 2.4. Histopathology

The randomly selected gastric mucosa samples from rats were collected and placed in the phosphate-buffered 10% formalin for histological assessment. After sample dehydration, the biopsies embedded in wax were sectioned at 5 *μ*m and stained with hematoxylin and eosin for histopathological examination by light microscopy.

### 2.5. Enzyme Linked Immunosorbent Assay (ELISA) Assessment

Plasma concentrations of *β*-endorphin, enkephalin, dopamine, melatonin, serotonin, cortisol, noradrenaline, and adrenaline were determined using ELISA kits purchased from BD Biosciences (San Jose, California, USA). The ELISA-based method was carried out according to the protocol provided by the manufacturer.

### 2.6. Biological Sample Collection and NMR Experiments

Sample preparation and data processing followed the methods of Xu et al. 2017 [19]. After the treatment course, the animals were killed by exsanguination under isoflurane anesthesia. For all animals, whole blood was drawn from carotid arteries using a catheter and left to clot at room temperature for 1h. Then, the samples were centrifuged at 12,000 ×g for 10 min at 4°C and then stored at -80°C until further ELISA study. In addition, the samples from cortex, stomach, and liver tissues were excised and snap frozen in liquid nitrogen and stored at -80°C until further metabolite extraction.

The aqueous metabolites in tissue samples were extracted according to the protocol reported by Wu* et al.* [23]. Prior to NMR analysis, 300 mg of tissue samples was homogenized with 1.40 mL of methanol and 0.56 mL of water and then vortexed for 60 s. After 10 min partitioning on ice, the samples were centrifuged for 5 min (12,000 ×g, 4°C). The supernatant layers were transferred into 2 mL tubes and lyophilized to remove the solvents.

The dried extracts were reconstituted with 500 *μ*L D_2_O containing 1 mM TSP and then transferred into 5 mm NMR tubes and analyzed by NMR spectroscopy. ^1^H NMR spectra of these samples were acquired at 296 K using a Bruker 600 MHz NMR spectrometer (Bruker Biospin, Germany) equipped with a cryogenic inverse detection probe. The NMR spectra were recorded using the NOESYPR1D (delay-90°-t_1_-90°-t_m_-90°-acquisition) pulse sequence. For each sample, 64 FIDs were collected into 64K data points over a spectral width of 12 000 Hz with a relaxation delay of 2s, a waiting time (t_1_) of 6.5 *μ*s, and a mixing time (t_m_) of 120 ms.

A series of 2D NMR spectra were acquired for peak assignments, which included ^1^H-^1^H correlation spectroscopy (COSY) [24], ^1^H-^1^H total correlation spectroscopy (TOCSY), ^1^H J-Resolved, ^1^H-^13^C heteronuclear single quantum correlation (HSQC), and heteronuclear multiple bond correlation spectra (HMBC). In addition, metabolites in tissue ^1^H NMR spectra were also assigned with reference to published data [25], HMDB database (http://www.hmdb.ca/), and an in-house NMR database.

### 2.7. Data Preprocessing and Multivariate Statistical Analysis

The acquired ^1^H NMR spectra were phase- and baseline-corrected using the MestReNova v 9.0.1 software (Mestrelab Research S.L.). Each spectrum was referenced to TSP at *δ* 0.00 and peak aligned manually to overcome peak-shift problem [26]. Then, the spectral regions which include the resonance from water (*δ* 4.70-5.20) and the baseline (peak-free) regions (*δ* 5.55-5.75, 6.25-6.50, 6.55-6.85, and 9.65-10.00) were removed for further analysis. The spectra over the ranges of *δ* 0.50-10.00 for tissue samples were binned into buckets with fixed width of *δ* 0.002. Prior to statistical analysis, the bucketed data were normalized by using the probabilistic quotient normalization method [27] to compensate for differences in overall concentrations of samples.

Next, the NMR spectral data were exported as Microsoft Excel files and imported into SIMCA software (version 14.1, Umetrics AB, Umea, Sweden) for multivariate analysis. The partial least squares discriminant analysis (PLS-DA) and orthogonal partial least squares discriminant analysis (OPLS-DA) were used to examine the metabolic difference due to electroacupuncture or moxibustion treatment. The validation of these models was performed with a seven-fold cross-validation and permutation test (200 permutations) [28]. Besides, the fold change and Student's* t*-test with a Bonferroni correction were used for univariate analysis. The resulting* t* statistic, such as transformed* p-*value, was used to determine statistical significance of differential metabolites in metabolomics. The fold change was expressed by the log ratio of concentrations between two conditions to identify significant metabolite changes above an arbitrary cut-off value. The concentrations of metabolites were quantified by integrals over corresponding spectral range in reference to the internal standards.

In the present study, both* t*-test and fold-change criteria were summarized in one single volcano plot. It is a scatter plot of* –*log_10_(*p*-value) against log_2_(fold change) [29]. The variable importance projection (VIP) and absolute correlation coefficient values (|*r*|) obtained from the OPLS-DA models were also integrated into the original volcano plot in the form of circles size and colour, respectively. In the plot, a larger circle size corresponds to larger VIP value and warmer colour corresponds to higher |*r*|. This enhanced four-dimensional volcano plot provides an intuitional way to identify potential biomarker candidates.

### 2.8. Pathway Analysis

The metabolite set enrichment analysis and pathway analysis were performed using the MetaboAnalyst v4.0 (http://www.metaboanalyst.ca) [30, 31]. In the analysis, the* Rattus norvegicus *(rat) pathway library was chosen, and the Fisher's exact test was selected for the overrepresentation analysis, and relative betweenness centrality was chosen for topology analysis.

## 3. Results and Discussions

### 3.1. Histopathological Examination of Gastric Mucosa

First, microscopic examinations were conducted to study the effects of electroacupuncture or moxibustion treatment on morphology of gastric mucosa. As shown in Figure S1, the control rats showed gastric mucosal enterocyte structure with well-arranged cells, continuous submucosa, and muscularis without inflammatory cellular infiltration. Comparable morphology was also observed for all treatment groups, showing undisturbed membrane structure of gastric mucosa following electroacupuncture or moxibustion stimulation. Previously, electroacupuncture treatment was found to exert therapeutic effects on gastric mucosa lesion [19]. The current results indicated that electroacupuncture and moxibustion treatments have no effect on morphology of gastric mucosa if the rats are in healthy condition.

### 3.2. Effects of Electroacupuncture or Moxibustion Stimulation on Plasma Hormone Concentrations

**Figure 2:**
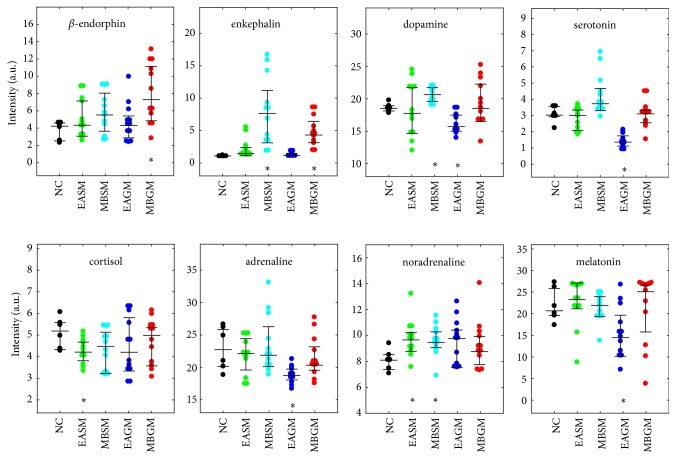
Changes in plasma hormone concentrations following electroacupuncture or moxibustion stimulation. Intensity of individual hormone is presented as colored dots according to different groups and three solid black horizontal lines represented the upper quartile, the median, and the lower quartile of its concentration, respectively. *∗ p*<0.05 comparing treatment groups with the control NC group.

The current results showed that plasma hormones concentrations are affected by both electroacupuncture and moxibustion treatments (Figure 2). Compared with the controls, groups treated with electroacupuncture on the GM acupoints showed lower levels of dopamine, serotonin, and adrenaline (all* p*<0.05). In contrast, the animals showed a lower cortisol but higher noradrenaline concentration by electroacupuncture treatment on the SM acupoints (all* p*<0.05). On the other hand, the groups which received moxibustion treatment had higher levels of *β*-endorphin and enkephalin following stimulation at the GM acupoints (all* p*<0.05), and higher levels of enkephalin, dopamine, and noradrenaline after treatment at the SM acupoints (all* p*<0.05).

It has been reported that electroacupuncture [32] or moxibustion [33] may lead to psychophysical responses, and stimulation through needling or mild heat on acupoints may activate receptors and secretion of neurotransmitters such as endorphins, serotonin, and noradrenaline [34]. In the current study, the plasma noradrenaline concentration was increased by both electroacupuncture and moxibustion stimulations at the SM acupoints. Previously, electroacupuncture stimulation on acupoints of the stomach meridian such as* Sibai* (ST2),* Liangmen *(ST21), and* Zusanli* (ST36) was found to enhance regularity of gastric myoelectrical activities and accelerate gastric emptying through the vagal pathway [19, 35]. The plasma noradrenaline concentration, which acts as indicator of autonomic function, has been reported to be proportional to the sympathetic activities [36]. It is documented that stimulation of the SM acupoints can improve gastric emptying and suppress the cyclooxygenase-2 (COX-2) protein expression in gastric tissues mediated via the sympathetic-COX-2 pathway [37]. Thus, the increased noradrenalin may be due to interaction between the sympathetic system and COX-2 activity.

On the other hand, the level of plasma enkephalin, which is a neurotransmitter involved in regulating nociception in the body [38, 39], was markedly increased following moxibustion on the SM or GM acupoints. Previously, Yi et al. [40] have reported the interaction between moxibustion signal and pain signal in the spinal cord, which may result in the local release of enkephalin. It is also suggested that plasma enkephalin is sensitive to thermal stimulation induced by moxibustion [41]. Taken together, the changes in plasma hormone levels observed in this study showed that electroacupuncture and moxibustion may serve as potential treatment techniques for hormone modulation.

### 3.3. ^ 1^H NMR Analyses of Tissue Extracts

Typical ^1^H NMR spectra of extracted cortex, stomach, and liver tissues are shown in Figure S2. Previously, we had shown that electroacupuncture simulations on different meridians exerted distinctive metabolic impact (i.e., meridian specificity) [42]. Here, the NMR data also showed significant effects on metabolic profiles perturbed by electroacupuncture or moxibustion stimulations on SM and GM acupoints, which are discussed in Sections 3.4 and 3.5, respectively.

### 3.4. Metabolic Responses due to Electroacupuncture or Moxibustion Stimulation on the Stomach Meridian

**Figure 3:**
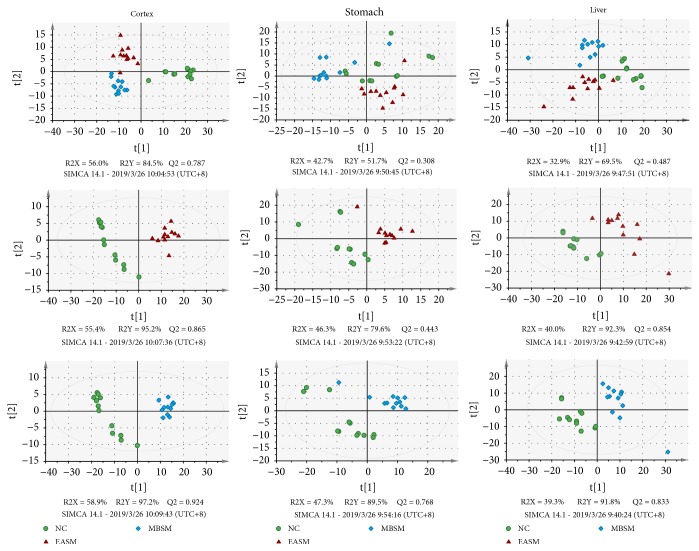
PLS-DA scores plots comparing the controls (NC) with electroacupuncture and moxibustion treatments on the SM acupoints, showing data from cortex (left column), stomach (middle column), and liver (right column) tissues.

*Multivariate analysis for stimulation on the SM acupoints*: to explore metabolic responses due to electroacupuncture or moxibustion intervention, PLS-DA was applied on the NMR data comparing the NC control group with the EASM and MBSM groups (Figure 3).

For both cortex and liver data, the controls separated well from the other two treatment groups in the first PLS-DA component (t[1]), indicating there are similar metabolic alterations due to treatments, irrespective of electroacupuncture or moxibustion stimulation. In addition, the EASM and MBSM group are separated in the second PLS-DA components (t[2]), which may be due to treatment-specific metabolic changes. Data from the stomach extracts also showed similar trend, but the group separation is less distinct. Pairwise comparison between the NC controls and each of treatment groups was also conducted and is shown in the second and third rows of Figure 3. The resulting models were found robust based on a seven-fold cross-validation and rigorous permutation test (200 permutations) (Figure S3).

**Figure 4:**
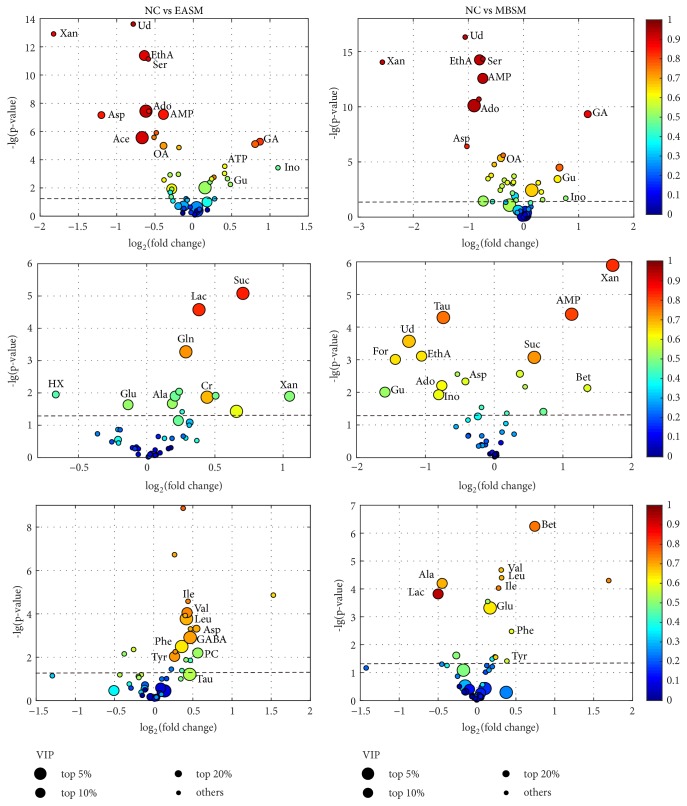
Enhanced volcano plots for screening of candidate metabolites associated with stimulation of the SM acupoints, showing data from cortex (top row), stomach (middle), and liver (bottom) tissues. VIP value and |*r*| are represented by circles size and colour, respectively. For each comparison, VIP values are categorized into four segments: top 5%, top 10%, top 20%. and the rest 80% with each represented by a circle of decreasing size. A cut-off dotted line that represents p-value = 0.05 (i.e., -lg(p-value)=1.30) is included in each plot, and differential metabolites with p < 0.05 will have value of -lg(p-value) > 1.30. Abbreviations: adenosine monophosphate (AMP), adenosine (Ado), adenosine triphosphate (ATP), acetate (Ace), alanine (Ala), aspartate (Asp), betaine (Bet), creatine (Cr), ethanolamine (EthA), formate (For), guanidinoacetate (GA), gamma-aminobutyrate (GABA), guanosine (Gu), glutamine (Gln), glutamate (Glu), hypoxanthine (HX), inosine (Ino), isoleucine (Ile), leucine (Leu), lactate (Lac), oxaloacetate (OA), phosphocholine (PC), phenylalanine (Phe), serine (Ser), taurine (Tau), tyrosine (Tyr), uridine (Ud), valine (Val), and xanthine (Xan).

**Figure 5:**
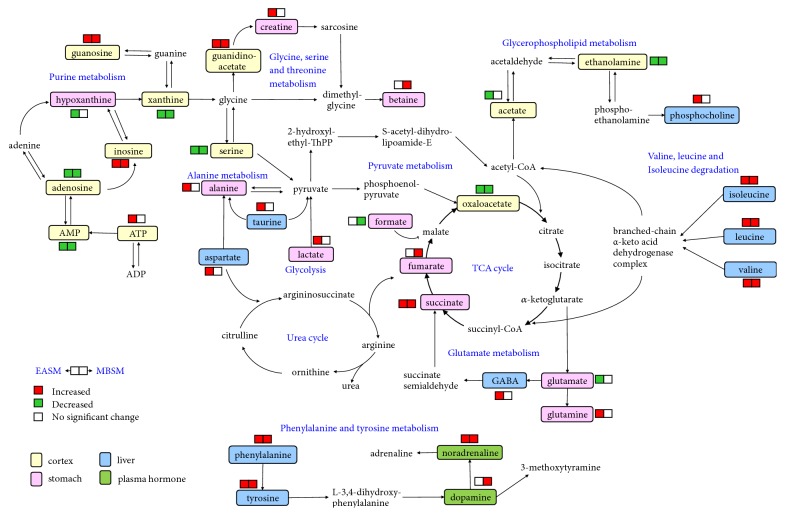
Metabolic perturbations due to electroacupuncture or moxibustion stimulation of the SM acupoints.

*Metabolic changes due to stimulations on the SM acupoints*: The group separation can be further improved by pairwise comparisons using the OPLS-DA (data not shown). The corresponding coefficient loadings and enhanced volcano plots (Figure 4) were then used to identify candidate metabolites that contributed to the intergroup separation. In this study, differential metabolites were selected based on the following criteria: the value of p < 0.05, correlation coefficient |*r*| ≥ 0.55, and VIP value ranked in the top 20% of total variables. The differential metabolites perturbed by stimulations on the SM acupoints are then mapped onto a metabolic map (Figure 5). In the figure, red and green boxes are used to represent the increased or decreased trends of metabolites, respectively.

The analysis highlighted a number of common metabolic changes following the electroacupuncture or moxibustion stimulation on the SM acupoints. In cortex, both treatments caused significant increase in the concentrations of guanidinoacetate (GA), inosine (Ino), and guanosine (Gu), together with decreased concentrations of ethanolamine (EthA), adenosine (Ado) monophosphate (AMP), adenosine (Ado), aspartate (Asp), and oxaloacetate (OA). This may be due to the intermediary role of brain cortex in the mechanism of acupoints stimulations [43]. In stomach, the common metabolic changes have been identified including succinate and xanthine. In addition, both electroacupuncture and moxibustion treatments were found to increase the concentrations of three branched-chain amino acids, i.e., valine (Val), leucine (Leu), and isoleucine (Ile) in liver.

The role of purines and purinergic signaling in acupuncture has been reported previously [44]. Purines are known to be key components in cellular energy systems [45]. In this study, the concentrations of nucleosides (e.g., hypoxanthine, xanthine, inosine, adenosine, guanosine, AMP, and ATP) were affected by both interventions. It has been suggested that mechanical deformation of the skin by needles or application of heat or electrical current leads to the release of large amount of ATP from keratinocytes, fibroblasts, and other cell types in skin [4, 46]. The released ATP then activates P2X3 ion channel receptors on sensory nerves within the skin that transmit messages via sensory ganglia and the spinal cord to the brain stem and hypothalamus. These brain regions contain motor neurons that control autonomic functions, including cardiovascular, gastrointestinal, respiratory, and urinogenital activities; all are common targets of acupuncture treatments [4]. Changes in concentrations of ATP, ADP, AMP, and adenosine had been reported in the neighboring interstitium following acupuncture at the SM* Zusanli *point in mice [47], which is consistent with our current findings.

Apart from the common metabolites, the stomach metabolome of the EASM group also showed higher concentrations of lactate (Lac), glutamine (Gln), creatine (Cr), and alanine, together with lower levels of tyrosine (Tyr) and hypoxanthine (HX) compared to the controls. In contrast, the MBSM group also showed significant increase levels of AMP and decrease levels of taurine (Tau), formate (For), ethanolamine, guanosine (Gu), uridine (Ud), adenosine, and inosine compared to the controls (Figure 4).

In liver samples, the levels of ethanolamine, glycerophosphocholine (GPC), phosphocholine (PC), taurine (Tau), and aspartate (Asp) were increased by the electroacupuncture treatment. In addition, the moxibustion treatment increased the levels of betaine (Bet) and glutamate (Glu) and decreased alanine (Ala) and lactate concentration.

Taken together, electroacupuncture treatment led to perturbations in amino acid metabolism including alanine, aspartate, and glutamine metabolism, as well as increased energy metabolism through higher production of ATP and lactate. Glutamate serves as a precursor for the synthesis of GABA, a neurotransmitter. This reaction is catalyzed by glutamate decarboxylase, which is most abundant in the cerebellum and pancreas. Conversion between glutamine, glutamate, and GABA maintains the internal balance between excitation and inhibition in CNS [48]. It has been reported that electroacupuncture activates a number of nonopioid pain-relevant neuronal systems in the central nervous systems operating via serotonin, noradrenaline, cholecystokinin, glutamate, and neuropeptide Y [49, 50]. In the EASM group, significant elevation of GABA and glutamine together with pronounced reduction of glutamate implies that electroacupuncture stimulation may exert excitement effects on CNS.

**Figure 6:**
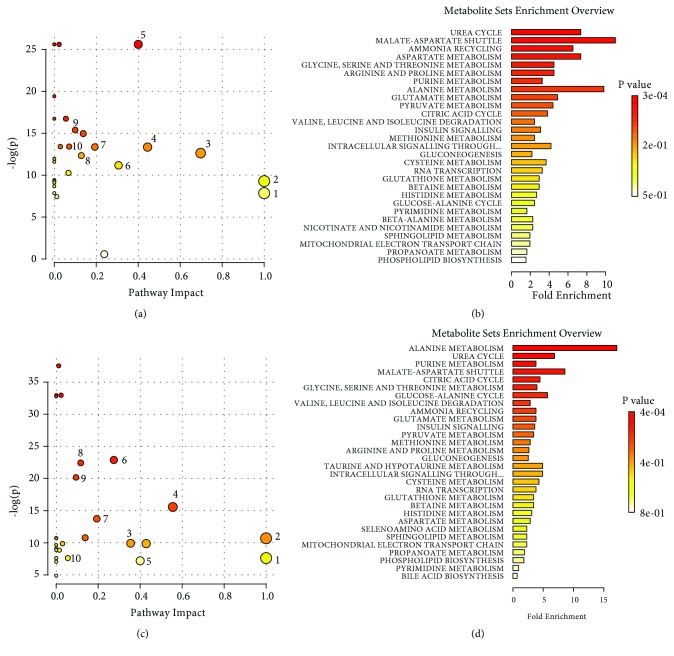
The pathway enrichment analysis for electroacupuncture and moxibustion stimulations on the SM acupoints. (a) Comparison between the NC and the EASM group, and its corresponding fold enrichment plot (b). (c) Comparison between the NC and the MBSM, and its corresponding fold enrichment plot (d). 1. Glutamate metabolism; 2. Valine, leucine, and isoleucine degradation; 3. Alanine metabolism; 4. Malate-aspartate shuttle; 5. Urea cycle; 6. Glycine, serine, and threonine metabolism; 7. Citric acid cycle; 8. Arginine and proline metabolism; 9. Purine metabolism; 10. Pyruvate metabolism.

*Pathway enrichment analysis for stimulations on the SM acupoints*: next, a metabolic pathway analysis for cortex, stomach, and liver extract samples was performed using MetaboAnalyst (Metabolomics Pathway Analysis) [30, 31]. As shown in Figure 6, electroacupuncture and moxibustion stimulations share a common set of perturbed metabolic pathways which included energy metabolism (such as malate-aspartate shuttle and citric acid cycle) and amino acid metabolism (including alanine metabolism, glutamate metabolism, glycine, serine and threonine metabolism, valine, leucine, and isoleucine degradation). Nevertheless, the rank of the perturbed pathways is different between EASM and MBSM groups.

### 3.5. Metabolic Responses to Electroacupuncture or Moxibustion Stimulation on the Gallbladder Meridian

**Figure 7:**
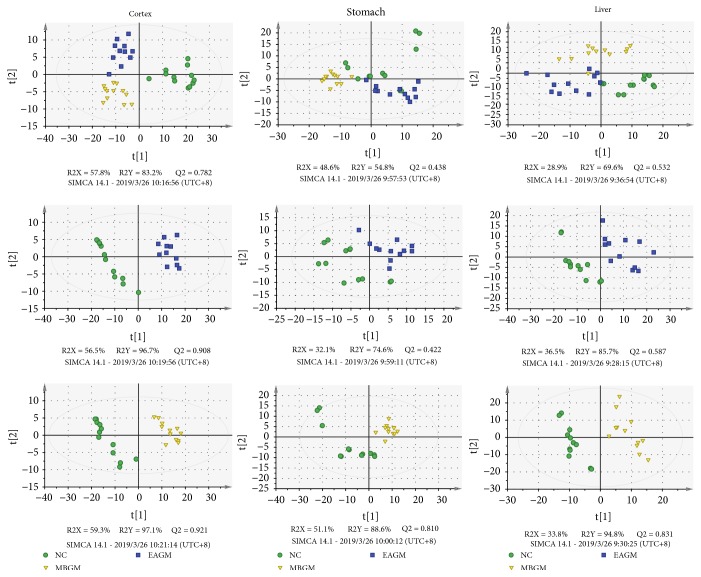
PLS-DA scores plots showing group separation due to the effect of stimulation on the GM acupoints for cortex (left column), stomach (middle column), and liver (right column) metabolome.

*Multivariate analysis for stimulations on the GM acupoints*: PLS-DA was performed on metabolomics data obtained from the NC, EAGM, and MBGM groups. For the cortex data, the PLS-DA score plots showed distinct separation among the EAGM, MBGM, and the control NC groups (Figure 7). The result suggested that the intervention-specific metabolic alterations are detectable in the metabolome of biological samples. On the other hand, mild group overlapping can be observed for the stomach and liver data.

When each of the treatment groups was compared with the controls, decent group separations can be achieved following pairwise comparisons (i.e., the NC and EAGM, NC and MBGM) using the PLS-DA (Figure 7) and OPLS-DA analysis. All models were found robust following a 7-fold cross-validation and permutation test (200 permutations) (Figure S4).

**Figure 8:**
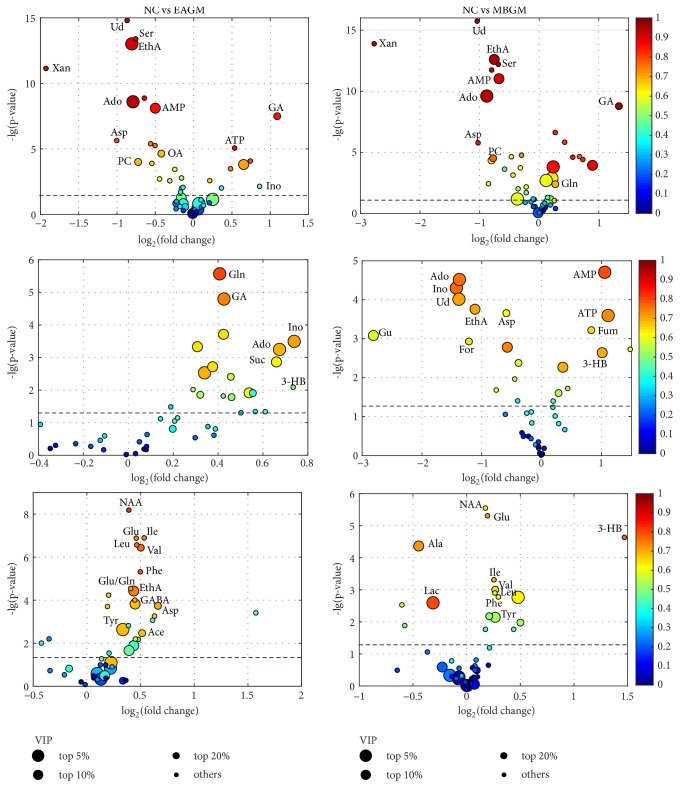
Enhanced volcano plots for screening candidate metabolites in stimulating the GM acupoints from cortex (top), stomach (middle), and liver (bottom) tissues. A cut-off dotted line that represents p=0.05 (-lg(p-value)=1.30) is included in each plot, and differential metabolites with p < 0.05 will have value of -lg(p-value) > 1.30.

**Figure 9:**
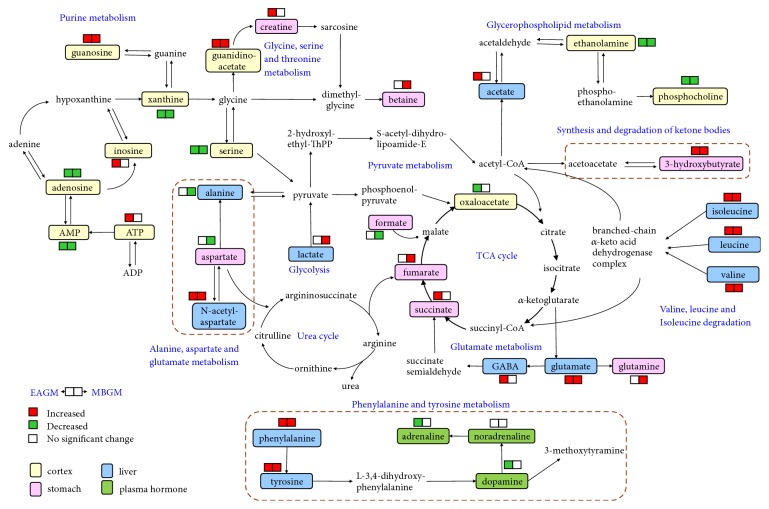
Summaries of metabolic pathways perturbed by stimulation of the GM acupoints. The meridian-specific changes are highlighted by the red dashed boxes.

*Metabolic perturbations for stimulations on the GM acupoints*: next, enhanced volcano plot (Figure 8) was used to identify differential metabolites due to electroacupuncture and moxibustion treatments on the GM acupoints. In addition, the differential metabolites were also mapped onto a metabolic map, as shown in Figure 9.

In comparison with the NC group, metabolic profile of cortex extracts in both EAGM and MBGM groups is highlighted with common changes including significant elevations of guanidinoacetate (GA) and reductions of ethanolamine, adenosine, AMP, xanthine, serine, and phosphocholine. Notably, most of these changes are found consistent for electroacupuncture and moxibustion stimulations on GM and SM acupoints.

For stomach extracts, significant higher levels of glutamine, guanidinoacetate, adenosine, inosine, succinate, and glutathione (GSH) were found following electroacupuncture stimulation on the GM acupoints. On the other hand, MBGM group showed increased concentration of AMP, ATP, and 3-HB and decreased adenosine, inosine, uridine, ethanolamine, and guanosine in stomach.

In liver, metabolic alterations in the EAGM group include increased N-acetyl-aspartate (NAA), glutamate, glutamate/glutamine, isoleucine, leucine, valine, phenylalanine, ethanolamine, and aspartate. On the other hand, elevated NAA, glutamate, GPC, and 3-HB and three branched-chain amino acid together with reduced alanine and lactate were associated with moxibustion stimulation on the GM acupoints.

Taken together, the EAGM and MBGM group showed metabolic changes in purine metabolism, glycine, serine and threonine metabolism, glycerophospholipid metabolism, valine, leucine and isoleucine degradation, pyruvate metabolism, TCA cycle, urea cycle, glycolysis, and glutamate metabolism. Previously, we had shown that meridian specificity is detectable at metabolome level [42]. Here, our current data also highlighted three pathways that were differentially perturbed due to stimulations at different meridian acupoints (Figures 5 and 9). These meridian-specific changes included alanine, aspartate and glutamate metabolism, synthesis and degradation of ketone bodies, and phenylalanine and tyrosine metabolism. Specifically, elevated N-acetylaspartate (NAA) was identified in the liver of both EAGM and MBGM groups. In addition, the concentration of 3-hydroxybutyrate (3-HB) was found increased in stomach extract following GM stimulation, which may be associated with increased fatty acid *β*-oxidation. On the other hand, both electroacupuncture and moxibustion stimulations on the SM acupoints, but not the GM acupoints, increased plasma noradrenaline concentration. These meridian-specific changes may contribute to the understanding of why stimulation on different meridian acupoints leads to distinctive therapeutic effects.

**Figure 10:**
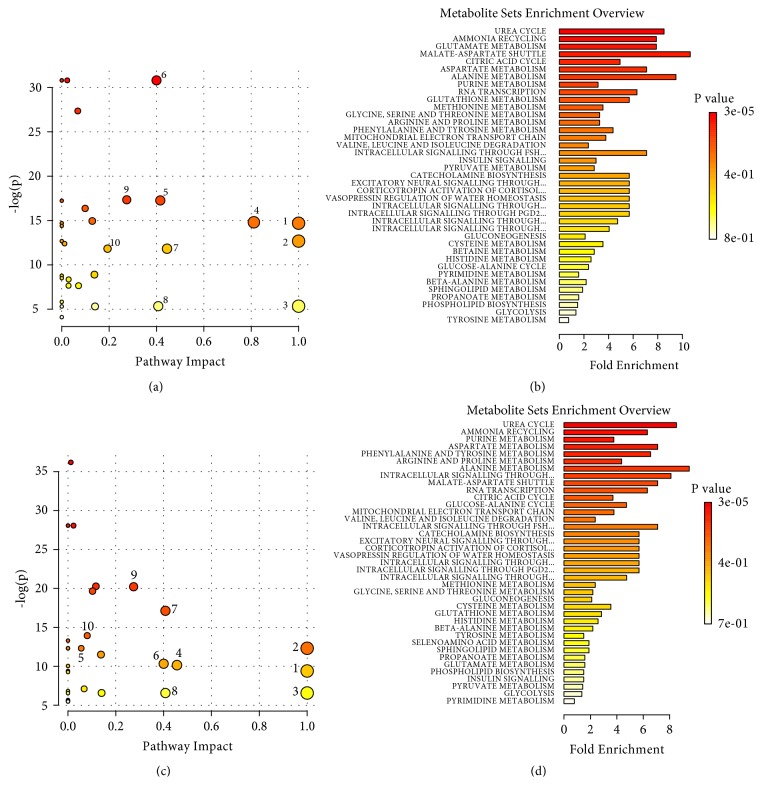
The pathway enrichment analysis for GM stimulation. (a) Comparison between the NC and the EAGM group, and its corresponding fold enrichment plot (b). (c) Comparison between the NC and the MBGM group, and its corresponding fold enrichment plot (d). 1. Valine, leucine, and isoleucine degradation; 2. Glutamate metabolism; 3. Phenylalanine and tyrosine metabolism; 4. Alanine metabolism; 5. Glutathione metabolism; 6. Malate-aspartate shuttle; 7. Purine metabolism; 8. Pyruvate metabolism; 9. Citric acid cycle; 10. Glycine, serine, and threonine metabolism.

*Pathway enrichment analysis for stimulation on the GM acupoints*: Similar trends of affected pathways were found in GM stimulation (Figure 10). With the combination of pathway topological analysis and fold enrichment results, top ten pathways containing input candidate metabolites were highlighted for their significant importance. Notably, most of the perturbed pathways are similar for both electroacupuncture and moxibustion stimulations

## 4. Conclusion

In the current study, metabolic effects of electroacupuncture and moxibustion on two classic meridians were examined using a healthy rat model. The metabolomic data highlighted common metabolic changes (irrespective of meridian and treatment), as well as treatment-specific and meridian-specific metabolic perturbations. The results exhibited an intricate interaction between meridian functionality and treatment methods at metabolic and hormonal levels. A comprehensive metabolomics study that includes all twelve known classic meridians may uncover metabolic mechanism of electroacupuncture and moxibustion and potentiate new applications beyond traditional practices.

## Data Availability

The data used to support the findings of this study are included within the article.

## Supplementary Materials

Supplementary Materials Supplementary Figure S1: photomicrographs of representative sections of gastric mucosa from five studied groups: (a) the control group, (b) electroacupuncture stimulation on SM acupoints, (c) moxibustion stimulation on SM acupoints, (d) electroacupuncture stimulation on GM acupoints, and (e) moxibustion stimulation on GM acupoints. Supplementary Figure S2: typical ^1^H NMR spectra of cortex, stomach, and liver extracts from the control rats. Supplementary Figure S3: permutation test for pairwise PLS-DA models comparing the controls with animals treated with the electroacupuncture (top row) or moxibustion (bottom row) stimulation on the SM acupoints. Supplementary Figure S4: permutation test for pairwise PLS-DA models comparing the controls with animals treated with the electroacupuncture (top row) or moxibustion (bottom row) stimulation on the GM acupoints.
