# Supplementary material for: Metabolic Response in Rats following Electroacupuncture or Moxibustion Stimulation
Source: Evid Based Complement Alternat Med. 2019 May 2;2019:6947471. doi: 10.1155/2019/6947471 (PMC6521395; doi:10.1155/2019/6947471)
Supplement: Supplementary Materials — Supplementary Figure S1: photomicrographs of representative sections of gastric mucosa from five studied groups: (a) the control group, (b) electroacupuncture stimulation on SM acupoints, (c) moxibustion stimulation on SM acupoints, (d) electroacupuncture stimulation on GM acupoints, and (e) moxibustion stimulation on GM acupoints. Supplementary Figure S2: typical 1H NMR spectra of cortex, stomach, and liver extracts from the control rats. Supplementary Figure S3: permutation test for pairwise PLS-DA models comparing the controls with animals treated with the electroacupuncture (top row) or moxibustion (bottom row) stimulation on the SM acupoints. Supplementary Figure S4: permutation test for pairwise PLS-DA models comparing the controls with animals treated with the electroacupuncture (top row) or moxibustion (bottom row) stimulation on the GM acupoints. [file 6947471.f1.pdf]

Supplementary Materials

Metabolic response in rats following electro-acupuncture or moxibustion stimulation

Jingjing Xu[a], Xianwei Lin[a], Kian-Kai Cheng[b], Huan Zhong[c], Mi Liu[c], Guoshan Zhang[c], Guiping Shen[a], Jiyang Dong[a,*]

[a] Department of Electronic Science, Fujian Provincial Key Laboratory for Plasma and Magnetic Resonance, Xiamen University, Xiamen 361005, China;

[b] Innovation Centre in Agritechnology, Universiti Teknologi Malaysia, 84600 Muar, Johor, Malaysia

[c] College of Acupuncture and Moxibustion, Hunan University of Chinese Medicine, Changsha 410007, China;

* Correspondence to: Prof. Jiyang Dong, Department of Electronic Science, Xiamen University, 422 Siming Nan Road, Xiamen 361005, China. Email: jydong@xmu.edu.cn, Fax: (+86) 592-2183301

Supplemental Figure S1. Photomicrographs of representative sections of gastric mucosa from five studied groups. (a) the control group, (b) electro-acupuncture stimulation on SM acupoints, (c) moxibustion stimulation on SM acupoints, (d) electro-acupuncture stimulation on GM acupoints, (e) moxibustion stimulation on GM acupoints.

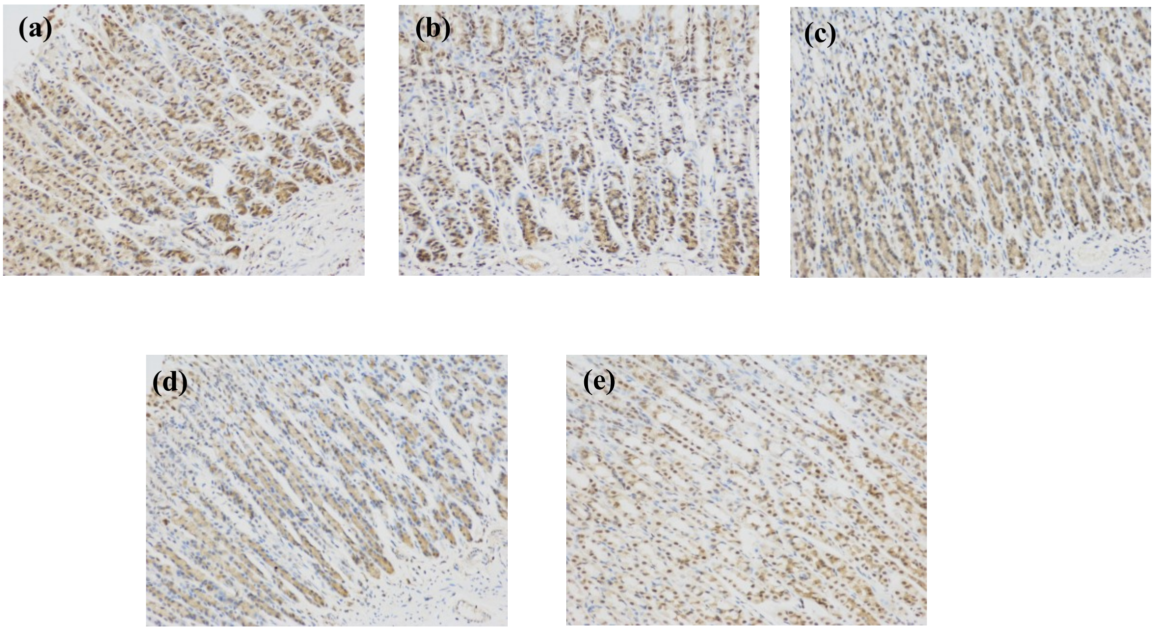

Supplemental Figure S2. Typical ^{1}H NMR spectra of cortex, stomach and liver extracts from the control rats.

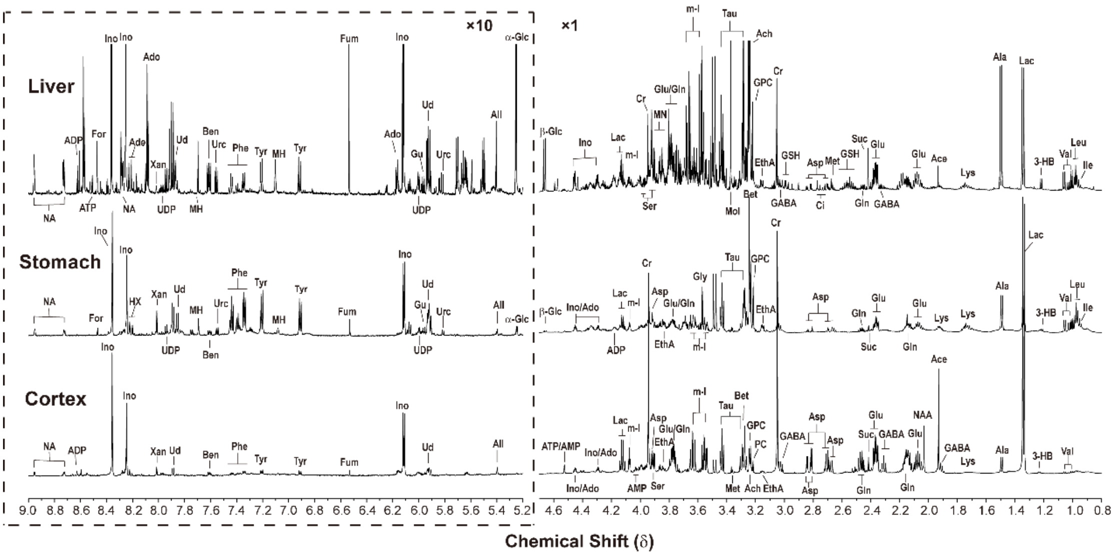

Abbreviations: Ace, acetate; Ach, acetylcholine; Ade, adenosine; Ado, Adenosine; ADP, Adenosine 3’,5’-diphosphate; Ala, alanine; All, allantoin; AMP, adenosine monophosphate; Asn, asparagine; Asp, aspartate; ATP, adenosine triphosphate; Bet, betaine; Ben, benzoate; Ci, citrate; Cr, creatine; DMA, dimethylamine; DMG, N,N-dimethylglycine; EthA, ethanolamine; For, formate; Fum, fumarate; GABA, gamma-aminobutyrate; Gln, glutamine; Glu, glutamate; Gly, glycine; GPC, glycerophosphocholine; GSH, glutathione; Gu, guanosine; 3-HB, 3-hydroxybutyrate; His, histidine; HPA, hydroxyphenylacetate; HX, hypoxanthine; Ile, isoleucine; m-I, myo-inositol; Ino, inosine; α-KG, alpha-ketoglutarate; Lac, lactate; Leu, leucine; Lys, lysine; MH, 1-methylhistidine; Mol, methanol; NA, nicotinamide; NAA, N-acetylaspartate; NAG, N-acetylglutamate; OA, oxaloacetate; PC, phosphocholine; Phe, phenylalanine; Ser, Serine; Suc, succinate; Tau, taurine; Tyr, tyrosine; Urc, urocanate; Ud, uridine; Val, valine; Xan, xanthine.

Supplemental Figure S3. Permutation test for pair-wise PLS-DA models comparing the controls with animals treated with the electro-acupuncture (top row) or moxibustion (bottom row) stimulation on the SM acupoints.

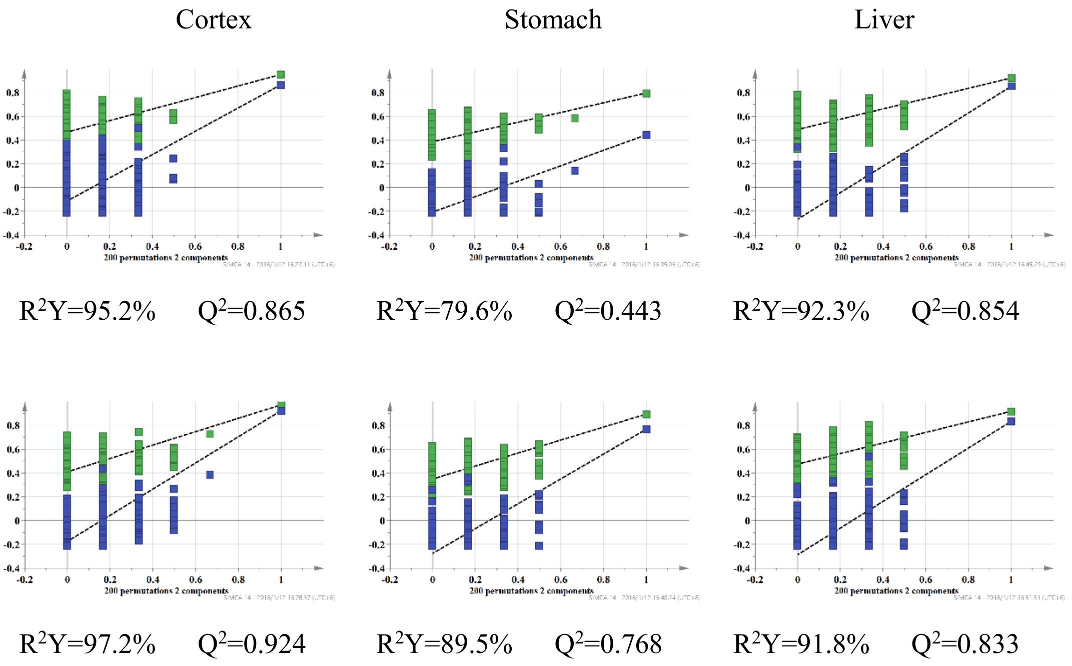

Supplemental Figure S4. Permutation test for pair-wise PLS-DA models comparing the controls with animals treated with the electro-acupuncture (top row) or moxibustion (bottom row) stimulation on the GM acupoints.

Cortex | Stomach | Liver

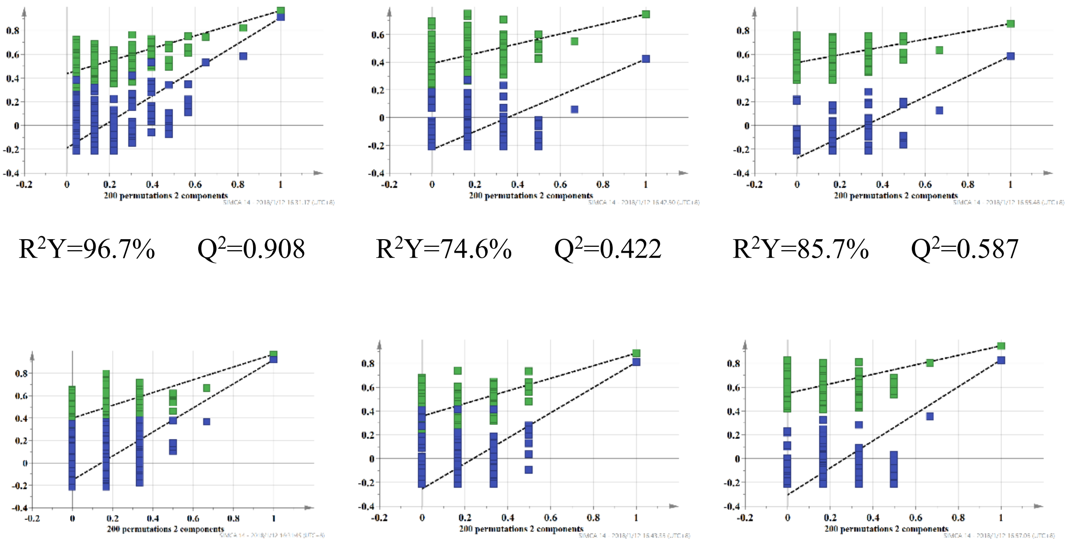

Top row: R^2Y=96.7% Q^2=0.908 | R^2Y=74.6% Q^2=0.422 | R^2Y=85.7% Q^2=0.587

Bottom row: R^2Y=97.1% Q^2=0.921 | R^2Y=88.6% Q^2=0.810 | R^2Y=94.8% Q^2=0.831
